# Supplementary material for: Functional Roles of LaeA-like Genes in Fungal Growth, Cellulase Activity, and Secondary Metabolism in Pleurotus ostreatus
Source: J Fungi (Basel). 2022 Aug 25;8(9):902. doi: 10.3390/jof8090902 (PMC9502681; doi:10.3390/jof8090902)
Supplement: Supplementary file 1 [file jof-08-00902-s001.zip › jof-1856782-supplementary.pdf]

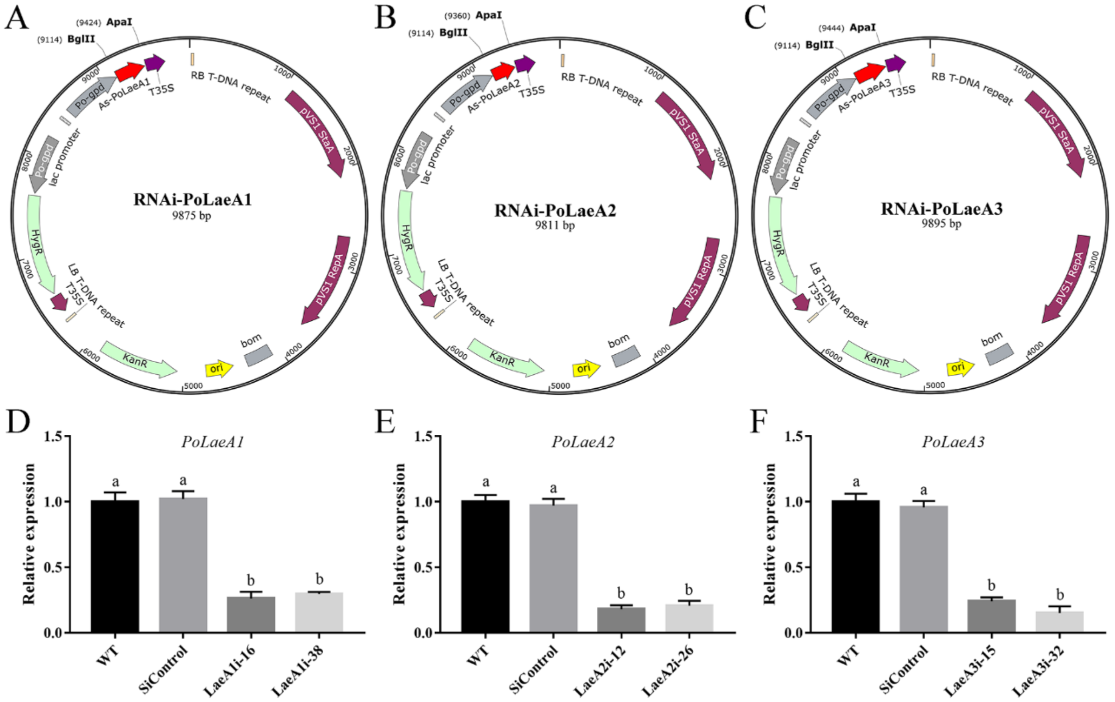

Figure S1. Construction of *PoLaeA*-silenced plasmids and strains. **(A–C)** Plasmid structure of RNAi-PoLaeA1 (**A**), RNAi-PoLaeA2 (**B**) and RNAi-PoLaeA3 (**C**). In these plasmids, the *HygR* gene is driven by the glyceraldehyde-3-phosphate dehydrogenase promoter of *P. ostreatus* (Po-gpd). The 300 bp, 236 bp and 320 bp antisense fragments of *PoLaeA1*, *PoLaeA2* and *PoLaeA3* were used to silence their expression, respectively. (**D**–**F**) qRT–PCR analysis of the expression of the *PoLaeA* genes. The expression levels of *PoLaeA1* (**D**), *PoLaeA2* (**E**) and *PoLaeA3* (**F**) in the WT strain were arbitrarily set to 1. The transformant with the empty vector pHPG was used as the control (SiControl). The values are the mean ± SD (n=3). Different lowercase letters indicate significant differences between the strains ($P < 0.05$, according to Dunnett's multiple comparisons test).

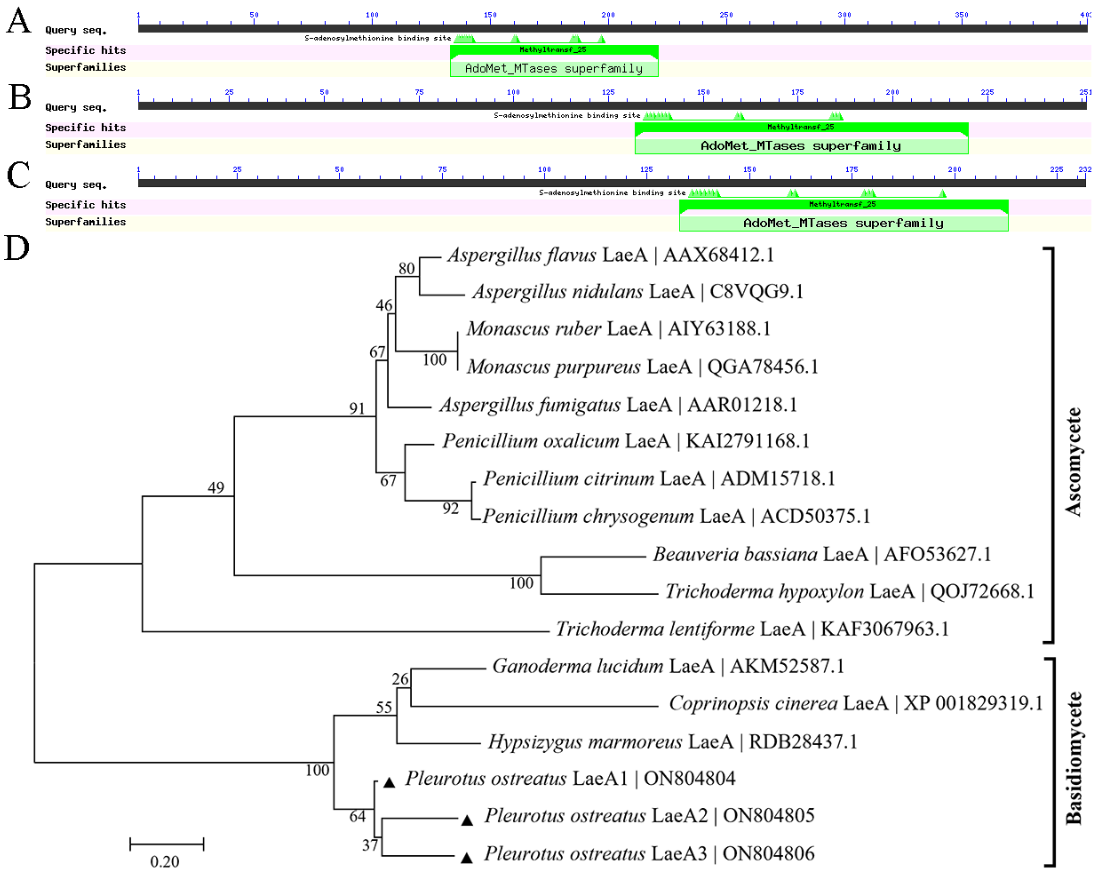

Figure S2. Protein domain structures and phylogenetic analysis of PoLaeA1, PoLaeA2 and PoLaeA3. (**A**–**C**) Protein domain structures of PoLaeA1 (**A**), PoLaeA2 (**B**) and PoLaeA3 (**C**). The conserved domains and S-adenosylmethionine (SAM) sites were identified by using the NCBI Conserved Domains database (https://www.ncbi.nlm.nih.gov/cdd, accessed on 16, May 2022). Asterisks indicate S-adenosylmethionine binding sites. (**D**) Phylogenetic relationship of LaeA-like proteins from different fungal species. The evolutionary history was deduced by the neighbor-joining method with 1000 replicates by MEGA 6.0.

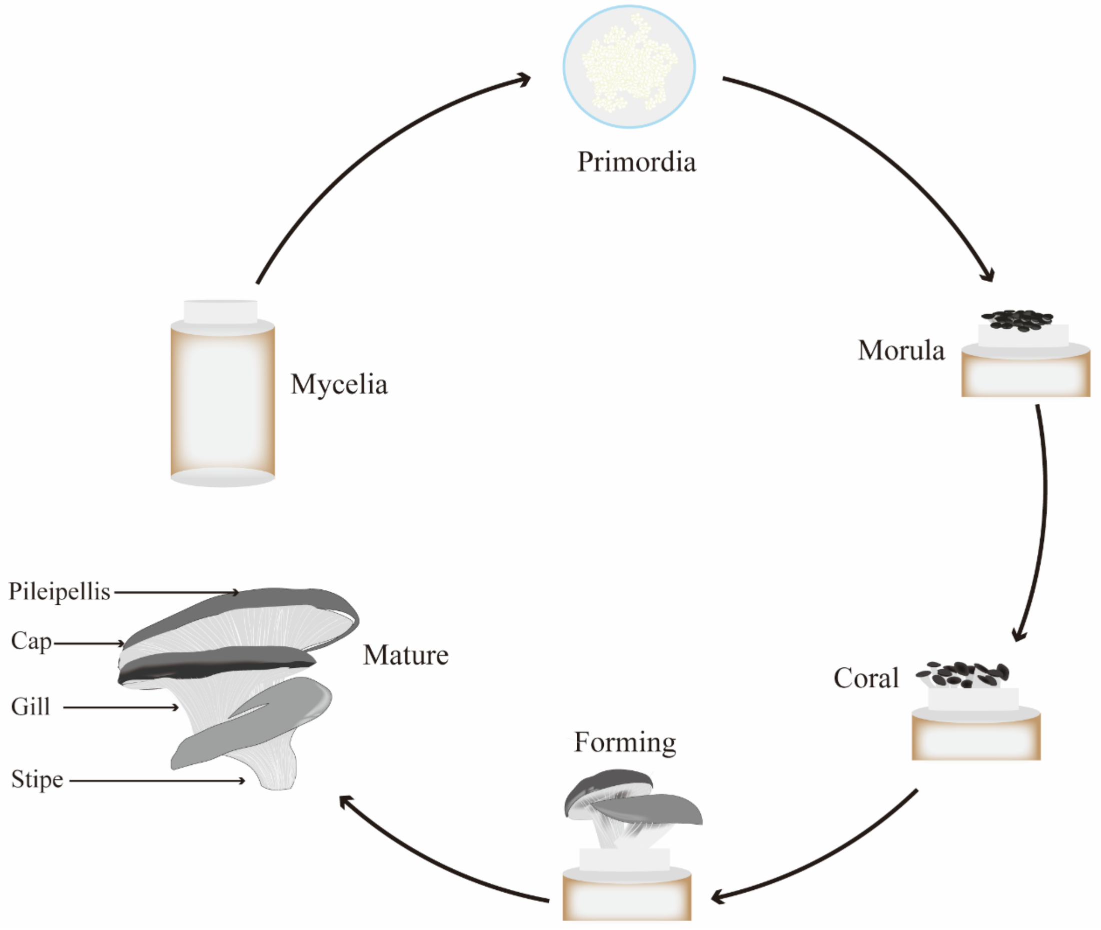

Figure S3. The diagram of the developmental phases and structures of *Pleurotus ostreatus*.

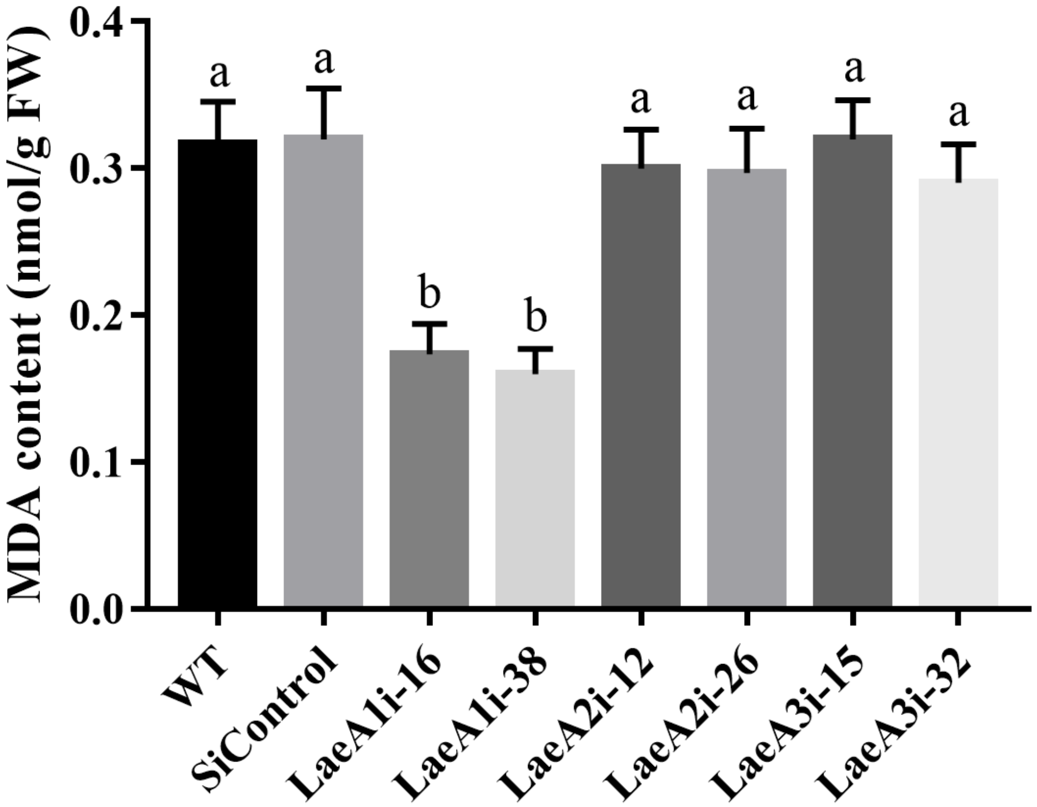

Figure S4. The malondialdehyde (MDA) content of the *PoLaeA*-silenced strains. The *P. ostreatus* strains were cultured in liquid CYM at 28 °C for 5 days. The values are the mean ± SD (n=3). Different lowercase letters indicate significant differences between the strains ($P < 0.05$, according to Dunnett's multiple comparisons test).

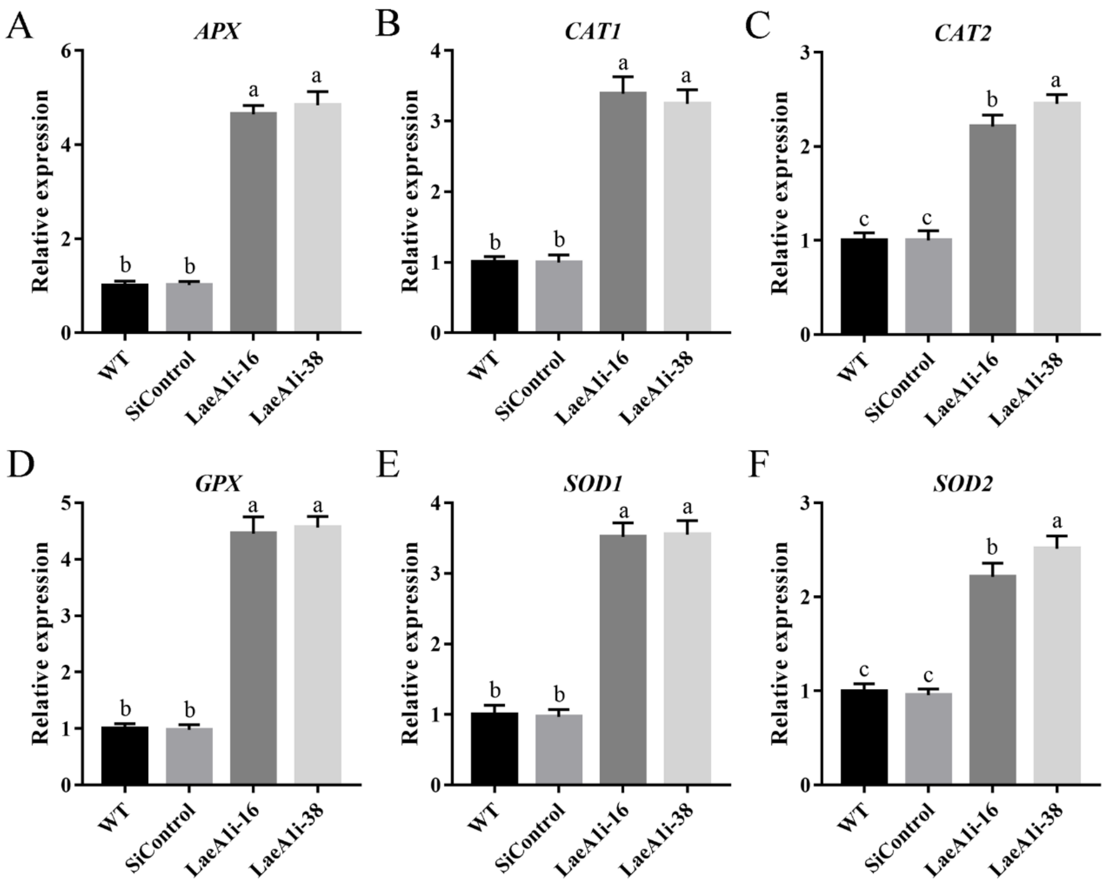

Figure S5. Transcriptional analysis of ROS-related genes in the *PoLaeA1*-silenced strains. The fungal strains were cultured in liquid CYM at 28 °C for 5 days. The expression levels of the ROS-related genes in the WT strain were arbitrarily set to 1.0. The values are the mean ± SD (n=3). Different lowercase letters indicate significant differences between the strains ($P < 0.05$, according to Dunnett's multiple comparisons test).

Table S1. Oligonucleotide primers used

Primer	Sequence (5′to 3′)	Description
PoLaeA1-F	ATGTCGACCGTACACAATAAT	Get the full length of *PoLaeA1*
PoLaeA1-R	CTATATTGTTAGGGACTGGAAC	
PoLaeA2-F	ATGTCCGTCGTCCCCCATAAT	Get the full length of *PoLaeA2*
PoLaeA2-R	TCAGCGGTTGCGTGTCCTC	
PoLaeA3-F	ATGTCGGTCGTGACCAATAAC	Get the full length of *PoLaeA3*
PoLaeA3-R	CTACAAAAATATTCCTCCTGAGA	
LaeA1i-F	GGAagatctAGTAGGTGCTTTAACGTG	Get the antisense silencing
LaeA1i-R	AgggcccCAAAGGTTCCAAGACTAG	fragment of *PoLaeA1*
LaeA2i-F	GGAagatctTGGTGGCTGAGCGTGTC	Get the antisense silencing
LaeA2i-R	AgggcccTTGCGGCGTCATTCTAT	fragment of *PoLaeA2*
LaeA3i-F	GGAagatctTGCTGCTACTGTACGCC	Get the antisense silencing
LaeA3i-R	AgggcccTCCCTTTCGACCTCATT	fragment of *PoLaeA3*
GAPDH-qF	GTGTTAACCTCGAGACTTACG	Detects the *GAPDH* expression
GAPDH-qR	TGGTGGCGTGGATTGTGCTC	
LaeA1-qF	TCCACCGTGTCCTCTT	Detects the *PoLaeA1* expression
LaeA1-qR	TAAATGCCGCTGCTTC	
LaeA2-qF	CACCGACTCGCACAGAT	Detects the *PoLaeA2* expression
LaeA2-qR	GCAGGTAGGCAATAGACAT	
LaeA3-qF	ACCACGGACTCGAACAG	Detects the *PoLaeA3* expression
LaeA3-qR	CAGGTAAGCCATAGACG	
Cch-qF	TGTTTCGTTCGGGCTAC	Detects the *Cch* expression
Cch-qR	GCAGAAGATACGGCACTC	
Mid-qF	TGTCCGTAGCTTTATGCG	Detects the *Mid* expression
Mid-qR	GAAACCCTCCCGTCCAG	
Yvc-qF	GCGATTCATCAAGAGTT	Detects the *Yvc* expression
Yvc-qR	TTTGTGAGGGTCGTAGA	
Plc-qF	GAAGTTCGTGGTATGGC	Detects the *Plc* expression
Plc-qR	CAAATGCTGTGAACCCT	
Cam-qF	GGAGGCGTTCAAGGTGT	Detects the *Cam* expression
Cam-qR	CATCGCCATCAACATCA	
Cna1-qF	GAAGGAAGAGTTGGAGGAG	Detects the *Cna1* expression
Cna1-qR	CACGAAGCAAAGCGAAT	
Cna2-qF	GCCATAGACGATACAGG	Detects the *Cna2* expression
Cna2-qR	TTCGCCTCCTTAGATAG	
Crz-qF	AGCCAAGGCATACTCAA	Detects the *Crz* expression
Crz-qR	GGAAATAGGAATCCGTTA	
Camk1-qF	TTCGGAGGTTCAAGGAG	Detects the *Camk1* expression
Camk1-qR	GGAGGTCTTCGCCAACT	
Camk2-qF	GAACAGAGGGAGCAGGAG	Detects the *Camk2* expression
Camk2-qR	GGCAGCCAAGAGTGATG	
Camk3-qF	CGATAAGATGGGAGACG	Detects the *Camk3* expression
Camk3-qR	ACTGCGGATTCAGGTGT	

Calreticulin-qF	TCCGACCCATCAAACAT	Detects the *Calreticulin* expression
Calreticulin-qR	CCGCTTTCCTCCACTTT	
CABP-qF	CGGAGTTTGGCAGAATA	Detects the *CABP* expression
CABP-qR	ACGCTCAGGGCACAGAT	
Ugp-qF	AGATAACCTTGGTGCCG	Detects the *Ugp* expression
Ugp-qR	CGAATAGAGCCCTCGTAG	
Pgm-qF	GCCGTACAGATCATCCT	Detects the *Pgm* expression
Pgm-qR	GACTAGCGGTCAACAAA	
Pgi-qF	CGTAGCGGCGAGTGGAA	Detects the *Pgi* expression
Pgi-qR	ACGAAGTGGGCGGTCAA	
Cbh1-qF	GGCTCCGACAACTGCTA	Detects the *Cbh1* expression
Cbh1-qR	GAGCGTCAGAGCGTTTC	
Cbh2-qF	CCGAAACCAAGTACCAGA	Detects the *Cbh2* expression
Cbh2-qR	GTTAGTGGAGAACTGAGCC	
Cbh3-qF	CGGACCTATCGGCTCTA	Detects the *Cbh3* expression
Cbh3-qR	ACACTGGGCGTATTTGG	
Cbh4-qF	GCTCGCGTGTCTACTTG	Detects the *Cbh4* expression
Cbh4-qR	TCCGTCAGCCTCCATCT	
Eg1-qF	GCGAAATACAAGGACAA	Detects the *Eg1* expression
Eg1-qR	TGAGAATGAACTGGGATG	
Eg2-qF	TGCCTGTTTCTTCCCTTCC	Detects the *Eg2* expression
Eg2-qR	GCGGTTTCCCTTGTCCC	
Eg3-qF	CCACAAGCCAACTCATC	Detects the *Eg3* expression
Eg3-qR	AGGTACTGGTGCATCTCA	
Eg4-qF	CTCTGCGTTGGTTGTCTT	Detects the *Eg4* expression
Eg4-qR	TTGGTTGGAGAAGTGGC	
Bgl1-qF	GTCGGTGTAGTGGCTCA	Detects the *Bgl1* expression
Bgl1-qR	TGCTGCTAGGGAAAGAA	
Bgl2-qF	TCCCTCCAAGACAAAGT	Detects the *Bgl2* expression
Bgl2-qR	GTCGCCGAATCTAACAC	
APX-qF	CTTGTGGACTCTTGGTGGTG	Detects the *APX* expression
APX-qR	TGTTGCGGAGATGGTCAG	
CAT1-qF	TGTGCATTGGTTGAGAGAGG	Detects the *CAT1* expression
CAT1-qR	TACGACGCTACAACTTCCG	
CAT2-qF	CTCTGTGGGAAAGAAAGT	Detects the *CAT2* expression
CAT2-qR	GAAGAAGATAGGCGTGTT	
GPX-qF	AAGGTCTTCAGGCATTGTAT	Detects the *GPX* expression
GPX-qR	GTGGTTACGCTCACAGAA	
SOD1-qF	CAGGCATTGAAGGTTGTC	Detects the *SOD1* expression
SOD1-qR	GCGTAGTTGAAGATGAGGG	
SOD2-qF	ACACGAAGCATCATCAGACCTA	Detects the *SOD2* expression
SOD2-qR	GAAGAGCGAGTGGTTGATATGG	

The sequences with lowercase letters agatct and gggccc indicate the *Bgl* II and *Apa*I restriction sites, respectively.
